# Unlocking the secrets of glucose metabolism reprogramming: the role in pulmonary diseases

**DOI:** 10.3389/fphar.2025.1551452

**Published:** 2025-08-13

**Authors:** Zhen Li, Shuo-Xuan Chen, Shuai Jiang, Yi-Nong Yang, Xi-Chan Yan

**Affiliations:** ^1^ School of Basic Medical Sciences, Hunan University of Medicine, Huaihua, Hunan, China; ^2^ School of Rehabilitation Medicine and Health, Hunan University of Medicine, Huaihua, Hunan, China; ^3^ Qiandongnan People’s Hospital, Affiliated Hospital of Guizhou Medical University, Kaili, Guizhou, China

**Keywords:** glucose metabolism reprogramming, pneumonia, acute respiratory distress syndrome, chronic obstructive pulmonary disease, lung cancer, pulmonary fibrosis

## Abstract

Metabolic reprogramming is the process by which cells adapt to different patterns of energy metabolism in response to the demands of the microenvironment for energy and biological macromolecules. Glucose serves as the primary energy source for cellular survival, and its metabolic pathways are intricately associated with cellular functional states. Recent studies have demonstrated that alterations in glucose metabolism, along with non-metabolic functions of metabolic enzymes and metabolites, play crucial roles in the development and progression of lung diseases under inflammatory conditions. This review summarizes the regulatory mechanisms of glucose metabolism across various pulmonary disorders and discusses the non-metabolic functions of glycolytic enzymes and metabolites in relation to disease pathogenesis. We aim to shine new light on the diagnosis and treatment of lung disease.

## 1 Introduction

Lung organ characteristics depend on susceptibility to disease (Cho and Stout-Delgado, 2020). The lungs are a highly elastic organ that provides a unique environment for gas exchange, bringing the inhaled air containing harmful substances and the circulating blood into proximity. Therefore, pulmonary microenvironmental homeostasis is affected by a variety of external stimuli, including micro-organisms, dust, pollen and various chemicals (Yang IA. et al., 2022; Natalini et al., 2023). Normally, the lungs can counteract these environmental stimuli with the help of a variety of host defense mechanisms. However, fulminant inflammation and the development of lung disease can result from prolonged exposure of the lungs to pathogenic antigens or highly virulent microorganisms. At present, there is still a need for further research into the mechanisms of inflammatory lung diseases.

Metabolism represents a fundamental characteristic of living organisms (Liu and Summer 2019). The biological processes involved in the absorption, utilization, and breakdown of substances within the lungs demand substantial energy input (Xu et al., 2021). Metabolic reprogramming is the process by which cells adopt different patterns of energy metabolism to survive in different environments (Vander Heiden et al., 2009). Glucose is widely recognized as the principal energy substrate for the majority of cell types (Galant et al., 2015). The reprogramming of glucose metabolism is crucial in lung disease (Huang et al., 2021; Zhong et al., 2023; Yegambaram et al., 2024). To date, three major metabolic pathways associated with glucose metabolism have been identified: glycolysis, the tricarboxylic acid (TCA) cycle, and the pentose phosphate pathway (Han et al., 2016; Paul et al., 2022). Nevertheless, the significance of the shift from oxidative phosphorylation (OXPHOS) to glycolysis in lung cells under pathological conditions remain to be fully elucidated.

Lactate is universally recognized as the terminal metabolite of cellular glycolysis (Vander Heiden et al., 2009). A growing number of studies have found that cells in a nonmonic state during disease can further exacerbate the disease by producing lactate through glycolysis, a process known as reprogramming of glucose metabolism (Machado et al., 2022; Deng et al., 2020; Zhu et al., 2022; Guarnieri et al., 2023; Lai et al., 2023). Similarly, to support their rapid growth and proliferation, lung cancer cells exhibit a preference for producing lactate through the glycolysis pathway (Cong et al., 2018). In addition, other studies have observed increased glycolytic activity in lung tissue and elevated blood lactate levels in patients with pneumonia and acute respiratory distress syndrome (ARDS) (Zhong et al., 2023; Yuan et al., 2022; Yu et al., 2018). Nevertheless, the significance of cell reprogramming of glucose metabolism in lung disease is unclear. Metabolic reprogramming in lung disease is known to be associated with specific changes in the activity of glycolytic enzymes as well as in glucose metabolites (Wang et al., 2022; Jiang et al., 2021; Zhang C. et al., 2022). Notably, glycolytic enzymes and glucose metabolites have also been demonstrated to possess signaling capabilities, exerting regulatory effects on multiple key processes involved in immune cell activation and programmed cell death (Seki and Gaultier, 2017). The biological targets of these glycolytic components are directly relevant to immune responses and tumorigenesis, as they can inhibit specific enzymes or induce covalent modifications of proteins, thereby altering protein function in disease contexts. The complexity of metabolic reprogramming is likely to eventually lead to new treatment approaches, which may have a significant impact on the pathogenesis of lung diseases. Therefore, to provide a basis for the development of new treatment strategies for lung diseases, we summarized the progress made in research on glucose metabolism reprogramming in lung diseases. Furthermore, by integrating both metabolic and non-metabolic functions, we will provide a systematic overview of the regulatory roles of key glucose metabolic enzymes and their associated metabolites, emphasizing their potential as therapeutic targets in lung diseases.

## 2 Glucose metabolism reprogramming and lung diseases

**FIGURE 1:**
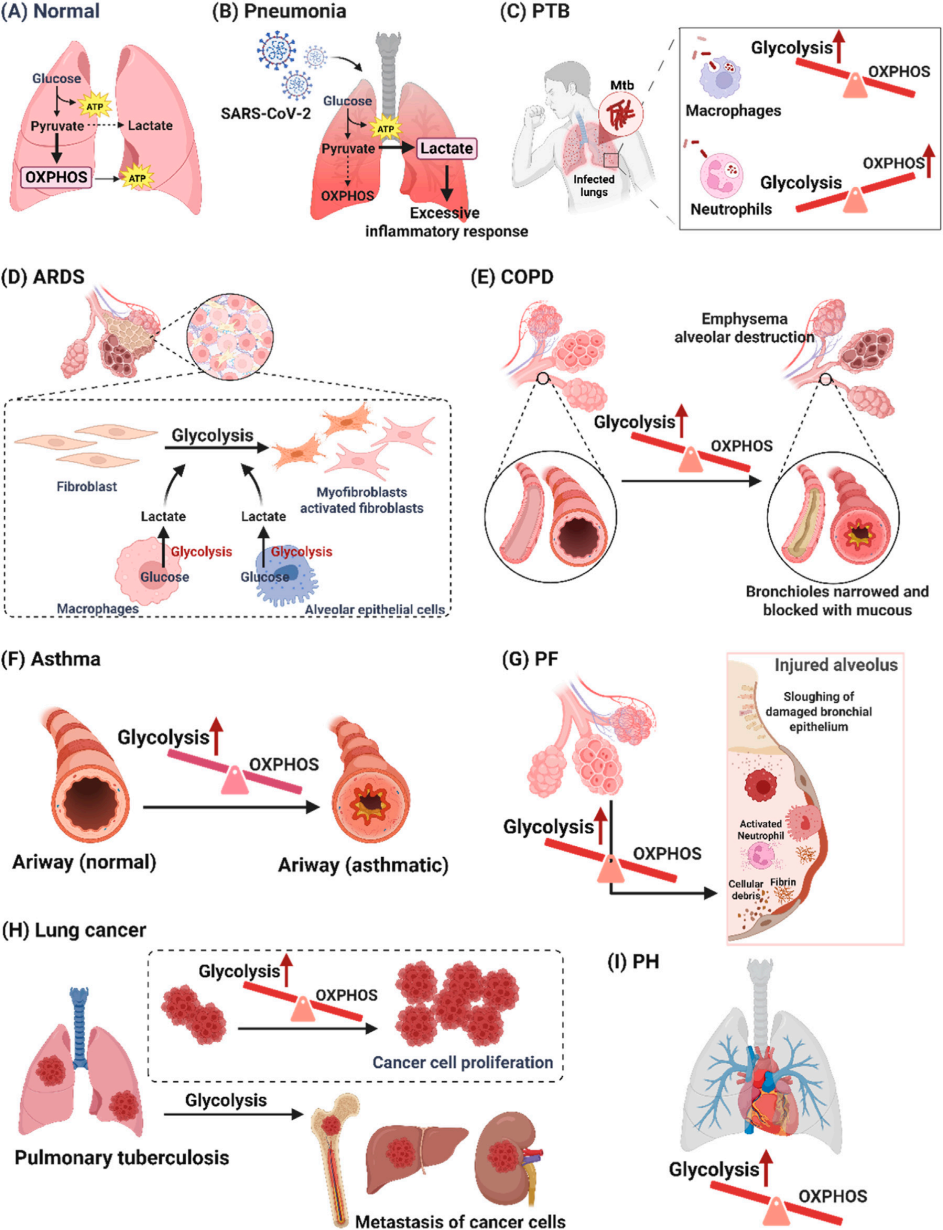
Glucose metabolism changes in pulmonary diseases. **(A)** Normally, glucose metabolism in the lungs is mainly OXPHOS. **(B)** The excessive inflammatory response is caused by increased glycolysis-dependent pathways in pneumonia. **(C)** The alterations in cellular metabolism induced by Mtb infection differed by cell type. Mtb infection resulted in enhanced macrophage glycolysis, whereas Mtb-infected neutrophils had elevated levels of mitochondrial metabolism and reduced glycolytic activity. **(D)** Increased glycolysis can exacerbate ARDS by activating the immune cells which release pro-inflammatory factors to damage epithelial cells. **(E)** Airway remodeling in COPD through a shift towards glycolysis in airway smooth muscle cells and bronchial epithelium. **(F)** Enhanced glycolysis exacerbates asthma, while reduced glycolysis relieves asthma. **(G)** Increased glycolysis triggers secreted lactate accumulation by alveolar epithelial cells and macrophages, which promotes the migration and activation of fibroblasts in PF. **(H)** Increased glycolysis can cause cancer cell growth and migration in lung cancer. **(I)** Glycolysis-mediated vascular cell dysfunction plays a crucial role in the pathophysiological process of PH. [PTB: pulmonary tuberculosis; Mtb: *mycobacterium tuberculosis*; ARDS: acute respiratory distress syndrome; COPD: chronic obstructive pulmonary disease; OXPHOS: oxidative phosphorylation; PF: pulmonary fibrosis; PH: pulmonary hypertension].

The pathogenesis of pulmonary diseases, including pneumonia, pulmonary tuberculosis (PTB), acute respiratory distress syndrome (ARDS), chronic obstructive pulmonary disease (COPD), asthma, pulmonary fibrosis (PF), lung cancer, and pulmonary hypertension (PH), involves alterations in glucose metabolic reprogramming. Based on the functional roles of glucose metabolism reprogramming in respiratory disorders, which are elaborated in the subsequent section, we have compiled a summary of the interplay between glucose metabolic reprogramming and lung diseases in Table 1, and illustrated the specific metabolic changes associated with each disease in Figure 1.

**TABLE 1:** Glucose metabolism reprogramming in pulmonary diseases.

Disease type	Metabolic pathway	Pathological trait	References
Pneumonia	OXPHOS to glycolysis	Increased inflammatory response	Guarnieri et al. (2023), Chelakkot et al. (2023)
PTB	—	Mtb infecting the lungs	Bagcchi, 2023; Shi et al. (2019), Andrews et al. (2024)
ARDS	OXPHOS to glycolysis	Diffuse alveolar injury and neutrophil-dominated inflammation	Jiang et al. (2024a), Roy et al. (2023)
COPD	OXPHOS to glycolysis	Emphysema (alveolar destruction Bronchioles Narrowed and blocked with mucous	Wang et al. (2023b)
Asthma	glycolysis	Chronic airway inflammation, airway hyperresponsive-ness, and reversible airflow limitation	Ledford et al. (2025)
PF	glycolysis	Extracellular matrix deposition and destruction of alveolar structure	Li et al. (2024a), Kim et al. (2023b)
Lung cancer	glycolysis	Rapid growth rate Susceptible to metastasis in the early stages	Yu et al. (2024), Chen et al. (2024b)
PH	OXPHOS to glycolysis	Endothelial cell dysfunction, abnormal proliferation of smooth muscle cells, vasoconstriction, inflammatory infiltration, and fibrosis of the tunica albuginea	Colon Hidalgo et al. (2022), Smolders et al. (2022)

### 2.1 Pneumonia

Glucose metabolism reprogramming is involved in various types of pneumonia. Community-acquired pneumonia (CAP) remains the leading cause of mortality among children under 5 years of age globally (Nair and Niederman, 2021). Under stress, cells alter glucose metabolism (e.g., increasing glycolysis-dependent pathways) to promote survival (Chelakkot et al., 2023). Recent research shows that activated glycolysis and elevated nucleotide levels disrupt deoxyribonucleotide balance, worsening CAP severity (Wang et al., 2023a). This metabolic dysregulation is closely associated with immune cell hyperactivation. Monocytes from CAP patients show increased expression of glycolysis-related genes and higher pyruvate levels (Otto et al., 2022), while neutrophils display elevated ATP, enhanced glycolysis, and inflammatory activation (Schuurman et al., 2023). Collectively, these findings indicate that excessive glycolysis contributes to inflammation in CAP. Viruses and bacteria are major causes of pneumonia. The H1N1 virus induces a shift toward glycolysis in alveolar epithelial cells (AECs), and blocking glycolysis reduces viral replication and inflammation (Meng et al., 2024). During *Mycoplasma pneumoniae* infection, bronchial epithelial cells show increased glucose uptake and enhanced glycolysis (He et al., 2024). Additionally, the peroxisome proliferator-activated receptor γ (PPAR-γ) agonist pioglitazone exerts a pro-inflammatory effect on bronchial epithelial cells during acute *P. aeruginosa* pneumonia by increasing intracellular glycolysis (Ferreira et al., 2022). Flagellin, a bacterial component, stimulates human bronchial epithelial cells to activate the mTOR pathway, increasing glycolytic flux and inflammatory factor secretion (Ramirez-Moral et al., 2021). Short-chain fatty acid (SCFA) acetate stimulates macrophages to increase their bactericidal activity through enhanced glycolysis (Machado et al., 2022). The findings point to glycolysis as a potential pharmacological target for the treatment of pneumonia by influencing immune cell activity and the secretion of pro-inflammatory factors.

Another specific pneumonia, known as coronavirus disease 2019 (COVID-19), has also recently been found to involve reprogramming glucose metabolism. COVID-19 is an acute respiratory inflammation caused by the SARS-CoV-2 virus infection. In one study, nasopharyngeal and autopsy tissue from patients with coronavirus disease was analyzed. The findings suggested that SARS-CoV-2 viral proteins are capable of interacting with host mitochondrial proteins, probably inhibiting OXPHOS and stimulating glycolysis, leading to severe COVID-19 pathology (Guarnieri et al., 2023). Cellular metabolic dysregulation is a consequence of SARS-CoV-2 infection, which is a key determinant of disease severity. By altering the glucose metabolism of immune cells, COVID-19 may affect the lung microenvironment. The study showed that mono-CD14^+^ cells expressed higher levels of glycolysis-related genes in severe COVID-19 patients than in mild patients (Qi et al., 2021). Meanwhile, a report examines the host immune and metabolic response pathways in patients with COVID-19-associated pulmonary mucormycosis (CAPM), suggesting that key pathways of glucose metabolism-glycolysis/gluconeogenesis were upregulated in monocytes from CAPM patients (Dhaliwal et al., 2024). In addition, another report showed that increased glycolysis and interleukin-6 signaling in cytotoxic T cells were significantly higher in patients with delayed severe COVID-19 compared to patients with usual severe COVID-19, particularly in the middle and late stages of infection (Kim IS. et al., 2023). The above studies suggest that dysregulation of immune cell glucose metabolism is closely related to the development of COVID-19. In parallel, based on the disruption of the lung microenvironment, it was shown that both fatty acid oxidation and hypoxia-induced mitochondrial respiration were converted to anaerobic glycolysis in CD8^+^ T cells, Natural killer T cells and epithelial cells from COVID-19 patients (Gurshaney et al., 2023). Clinical data also suggest that implementation of a ketogenic diet can reduce glycolytic activity in CD4^+^ lymphocytes, prolong survival, decrease hospitalization requirements, and exert protective effects against metabolic disturbances in patients with COVID-19 (Boleslawska et al., 2023). Meanwhile, another study found that glycolysis is essential for virus replication in a model of lung epithelial cell infection and that blocking glycolysis in the cell caused a significant reduction in virus production (Krishnan et al., 2021). The results suggest that COVID-19 may directly or indirectly cause abnormalities in glucose metabolism in lung structural cells, thereby accelerating the disease process.

### 2.2 PTB

PTB is a chronic respiratory infectious disease caused by *Mycobacterium tuberculosis* (Mtb) infecting the lungs, and its main symptoms include coughing, hemoptysis, fever, lethargy, and loss of appetite (Bagcchi, 2023). Macrophages are a highly heterogeneous group of cells in the body’s immune system, which play an important role in maintaining physiological homeostasis and immune regulation (Ahmad et al., 2022). Glycolysis is one of the main ways for macrophages to obtain energy. In transcriptome sequencing of bone marrow-derived macrophages from Mtb-infected mice, it was shown that Mtb infection resulted in increased expression of the key glycolytic enzymes (Shi et al., 2019). However, Mtb can suppress the immune response of macrophages by reducing lactate and IL-1β production through metabolic reprogramming of macrophages (C et al., 2021). The reprogramming of macrophage energy metabolism induced by Mtb infection reveals a complex relationship between host and pathogen (C et al., 2021). Neutrophils infected with Mtb are frequently found in the airways of patients with active tuberculosis, and excessive aggregation of neutrophils in the lungs has been associated with an increased bacterial load of tuberculosis and exacerbation of pathology. Studies have reported elevated levels of mitochondrial metabolism and reduced glycolytic activity in neutrophils from Mtb-infected lungs, and activated neutrophils carry more viable Mtb (Andrews et al., 2024). In recent years, the research on the mechanism of cellular energy metabolism regulation in Mtb infection has shown a wide and deep trend, and comprehensively revealing the effect of Mtb infection on cellular metabolism can provide a reference for the treatment and prevention of tuberculosis.

### 2.3 ARDS

ARDS is a high-mortality clinical syndrome characterized by diffuse alveolar damage (DAD). Glycolysis plays an important role in the development of ARDS by regulating macrophage polarization through metabolic reprogramming. The study suggests that hypoxia robustly stabilizes hypoxia-inducible factor-1α (HIF-1α) in tissue-resident alveolar macrophages (TR-AMs) to promote a glycolytic phenotype that exacerbates ARDS (Woods et al., 2022). Another study showed that the triggering receptor expressed on myeloid cell-1 (TREM-1) activation increased glucose consumption, induction of glycolysis, and inhibition of OXPHOS during ARDS (Zhong et al., 2023). The above studies have indicated that increased levels of glycolysis in macrophages exacerbate ARDS.

Stem cell engineering, glycolysis inhibitors, and traditional Chinese medicine are primarily employed by current therapeutic strategies targeting glycolysis reprogramming in ARDS. Apoptotic bodies released by transplanted human umbilical cord MSCs induce reprogramming of macrophage metabolism, which shifts from glycolysis to mitochondrial OXPHOS unequivocally ameliorates ARDS (Jiang T. et al., 2024). Exosomes secreted by bone marrow mesenchymal stem cells were able to modulate macrophage M1 polarization by inhibiting cellular glycolysis (Deng et al., 2020). Apelin-13 protects against lipopolysaccharide (LPS)-induced inflammation and ARDS by regulating glycolysis and modulating redox homeostasis in macrophages (Yuan et al., 2022). Pelitinib and Iso-seco-anpirtoline protect against ARDS by blocking JAK3-mediated glycolysis and pyroptosis in macrophages (Jiang W. et al., 2024; Kong et al., 2023). HIF-1α-mediated glycolysis represents a critical pathway contributing to inflammatory activation in ARDS, and both Dachengqi Decoction and N-phenethyl-5-phenylpicolinamide have been shown to exert therapeutic effects by inhibiting this pathway (Shang et al., 2024; Du et al., 2022). Phloretin is protected against LPS-induced ARDS by inhibiting glycolysis in macrophages via a glucose transporter 1 (GLUT1)-dependent pathway (Songyang et al., 2022). These findings suggest a potential pharmacological target for the treatment of ARDS by targeting glycolysis-exacerbated immune cell activation.

### 2.4 COPD

COPD is a set of progressive lung diseases characterized by continuous airflow limitation. COPD is closely linked to recurrent cycles of inflammation and infection, with dysregulated immune responses playing a key role in disease progression. Cigarette smoke (CS) exposure represents the primary risk factor for both the onset and progression of COPD. Research has demonstrated that CS exposure leads to alterations that switch to glycolysis via growth differentiation factor 15 (GDF15)-related pathways in the human bronchial epithelium (Wang et al., 2023b). The evidence has implied that when exposed to CS, AM tends to switch to high levels of glycolysis to provide the energy needed to produce inflammatory factors (Mei et al., 2022). Meanwhile, CS also inhibited mitochondrial respiration in model human acute monocytic leukemia cell and peripheral blood monocyte-derived macrophages, while inducing glycolysis and reactive oxygen species (Ar et al., 2019). The hedgehog interacting protein (HHIP) locus has been consistently associated with susceptibility to COPD. One study showed that HHIP deficiency increases reprogramming of glucose metabolism in airway smooth muscle cells following exposure to CS, thus contributing to airway remodeling in the pathogenesis of COPD (Li et al., 2021). In addition, a decrease in respiration during glucose metabolism was observed in the CS-exposed ATII cells, indicating a shift toward glycolysis (Agarwal et al., 2014). These results suggest that by influencing the pro-inflammatory response of immune cells and directly altering the function of lung structural cells, CS-induced reprogramming of glucose metabolism is involved in the pathophysiology of COPD.

### 2.5 Asthma

Bronchial asthma, abbreviated as asthma, is a heterogeneous disease characterized by chronic airway inflammation, airway hyperresponsiveness, and reversible airflow limitation (Ledford et al., 2025), with clinical manifestations including recurrent episodes of wheezing, shortness of breath, chest tightness, or coughing (Bacharier and Jackson, 2023). Glycolysis is closely linked to asthma, and a series of studies have shown that enhancing glycolysis exacerbates asthma, while reducing glycolysis relieves asthma.

Studies have shown that alveolar macrophages preferentially convert metabolism to glycolysis for energy production during the onset of asthma. It has been shown that formaldehyde exposure causes exacerbation of allergic asthma with infection by inducing glycolysis. Moreover, macrophage glycolytic genes and lactate secretion levels are upregulated in formaldehyde-exposed asthmatic mice and asthma patients, and the glycolysis inhibitor 2-DG can significantly improve formaldehyde -exposure-induced exacerbation of allergic asthma (Xuan et al., 2024). The proteins S100A8 and S100A9 can promote macrophage dysfunction and glycolysis. Overexpression of S100A9 can exacerbate lung injury and inflammation in patients with allergic asthma, whereas inhibition of S100A8 and S100A9 can stabilize macrophage polarization and inhibit glycolysis to improve allergic asthma (Ji et al., 2024). Treatment with dexamethasone significantly downregulates ovalbumin-induced glycolysis levels in THP-1 cells and modulates subsequent protein lactylation and NLRP3-mediated classical pyroptosis, thereby treating eosinophilic asthma in mice (Chen N. et al., 2024). The above study suggests that increased levels of glycolysis in macrophages exacerbate asthma. The transformation of airway smooth muscle cells (ASMCs) into a hyperfictional synthetic phenotype such as proliferation and secretion, leading to thickening of the airway smooth muscle layer, which is a key pathological mechanism of airway remodeling in severe asthma and is strongly correlated with the severity of asthma (Camoretti-Mercado and Lockey, 2021). A series of studies have shown that increases or decreases in glycolysis affect the proliferation, migration and function of ASMCs. Inhibition of glycolysis attenuated ATP production and bronchodilator-induced cAMP concentration in human airway smooth muscle cells and improved cell shortening (Xu S. et al., 2023). p62 enhances glycolysis to promote the proliferation and migration of bladder smooth muscle cells through activation of HK2 (Yu et al., 2021).

Glycolysis, a key pathway of cellular energy metabolism, plays a crucial role in the pathophysiology of asthma. Therefore, an in-depth exploration of the relationship between glycolysis and the inflammatory phenotype of asthma will not only contribute to a better understanding of the pathogenesis of asthma but also help to reveal the key factors that influence the progression and exacerbation of asthma.

### 2.6 PF

PF is a chronic progressive fibrotic disease characterized by deposition of extracellular matrix and destruction of alveolar structures, ultimately leading to respiratory failure. PF usually results from abnormalities in the alveolar structures caused by chronic exposure to toxic particles or gases. Based on this, we have summarized the role of reprogramming glucose metabolism in various drug-induced PF models. Airborne particulate matter (PM2.5) can cause lung inflammation and even fibrosis. One study showed that PM2.5 exposure induced increased glycolysis and changes in histone acetylation in macrophages, which exacerbated PF (Li J. et al., 2024). Chronic exposure to Cigarette smoke (CS) also contributes to PF by increasing fibroblast glycolysis to promote fibroblast activation (Li Q. et al., 2024). In another study, LPS was shown to increase fibroblast activation by stimulating aerobic glycolysis during sepsis-associated PF (Zhong et al., 2024). Lactate accumulation in PF tissue is a significant factor aggravating PF development. The evidence showed that bleomycin (BLM)-induced PF is associated with lactate accumulation due to upregulated glycolysis in AECs (Sun et al., 2024). Inhalation of silica causes the occupational disease silicosis, which typically results in progressive fibrosis of lung tissue. Research has shown that HIF-1α and glycolysis-related genes are upregulated in AMs after silica exposure, whereas PF is rescued by inhibiting glycolysis (Lu et al., 2024). In addition, the report found that glycolysis was also increased in macrophages from patients with post-COVID pulmonary fibrosis (Kim Y. et al., 2023). To summarize, external irritants exacerbate the disease by promoting reprogramming of glucose metabolism in various lung cells, which in turn affects the formation of chronic inflammation and fibrosis in PF.

### 2.7 Lung cancer

Lung cancer is the most common cause of cancer mortality around the world. Glucometabolic reprogramming has been recognized as a critical mechanism that contributes to the initiation and progression of tumors. Non-small cell lung cancer (NSCLC) is estimated to account for 80%–85% of all lung cancers, with lung adenocarcinoma (LUAD) being the most common histological subtype. Chaperonin-containing TCP1 subunit 6 A can facilitate the transcription of hexokinase 2 (HK2), a critical enzyme in glycolysis, thereby promoting glycolysis and progression of LUAD (Yu et al., 2024). Ubiquitin-specific protease 54 (USP54) inhibits glycolysis and tumor cell growth by reducing p53-mediated GLUT1 expression and ameliorates the malignant phenotype and poor survival of LUAD patients (Chen L. et al., 2024). Metastasis is an important contributor to increased mortality rates in NSCLC. It is noteworthy that the transforming growth factor-β1 (TGF-β1) signaling pathway is a key driver of tumor metastasis through epithelial-mesenchymal transition (EMT). Pirfenidone inhibits glycolysis during EMT in epithelial cells by targeting TGF-β1, thereby enhancing epithelial cell chemo-sensitization (Zhang S. et al., 2024). Another lung cancer that is strongly associated with alterations in glycolysis-related genes is lung squamous cell carcinoma (LUSC). This is one of the most common malignancies, and the Cancer Genome Atlas identified and validated for the first time that glycolysis is highly associated with the development of LUSC (Kadasah, 2024). In murine lung cancer models and a human LUSC patient-derived xenograft model, DNA-pharmacokinetics-mediated cytoplasmic DNA sensing enhanced glycolysis to improve LUSC cell viability, motility and chemoresistance (Wang H. et al., 2024). In summary, in advanced lung cancer, tumor tissue forms a microenvironment in which tumor cells are converted to glycolysis, directly or indirectly promoting tumor growth and migration.

### 2.8 PH

PH refers to a state of hemodynamic abnormality in which the pressure in the pulmonary arteries is elevated above a certain threshold, which can further develop into right heart failure and even death if not effectively controlled (Mocumbi et al., 2024). The main pathological features of PH include endothelial cell dysfunction, abnormal proliferation of smooth muscle cells, vasoconstriction, inflammatory infiltration, and fibrosis of the tunica albuginea (Colon Hidalgo et al., 2022). Endothelial cell dysfunction mediated by glycolysis plays a crucial role in the pathophysiological process of PH (Piper et al., 2024; Luo et al., 2024). The mRNA expression levels of *Glut1, Hk*, and lactate dehydrogenase metabolizing enzymes, which are key genes for glycolysis, were significantly increased in PH pulmonary vascular endothelial cells cultured *in vitro* (Smolders et al., 2022). The expression and activity of PFKFB3 were significantly increased in pulmonary vascular endothelial cells from PH patients and rodent models, and knockdown of PFKFB3 in endothelial cells attenuated vascular smooth muscle cell proliferation, endothelial injury and inflammatory cell recruitment in PH (Cao et al., 2019). Proliferation of pulmonary arterial smooth muscle cells (PASMCs) is central to pulmonary vascular remodeling in PH. In idiopathic PH, the mode of energy production in PASMCs is shifted from mitochondrial oxidative phosphorylation to glycolysis (Perros et al., 2019). The glycolysis inhibitor 3-bromopyruvic acid can improve the hemodynamic indices of hypoxia-induced PH. 3-bromopyruvic acid can inhibit the increased expression of HK2 and the elevated lactate concentration in PASMCs, thereby attenuating the proliferation and migration of PASMCs and effectively reversing PH-associated pulmonary vascular remodeling (Chen et al., 2018). Taken together, metabolic switching with glycolysis as the main form of energy supply may drive the development of PH.

## 3 Glucose metabolism reprogramming in various pulmonary cells

**FIGURE 2:**
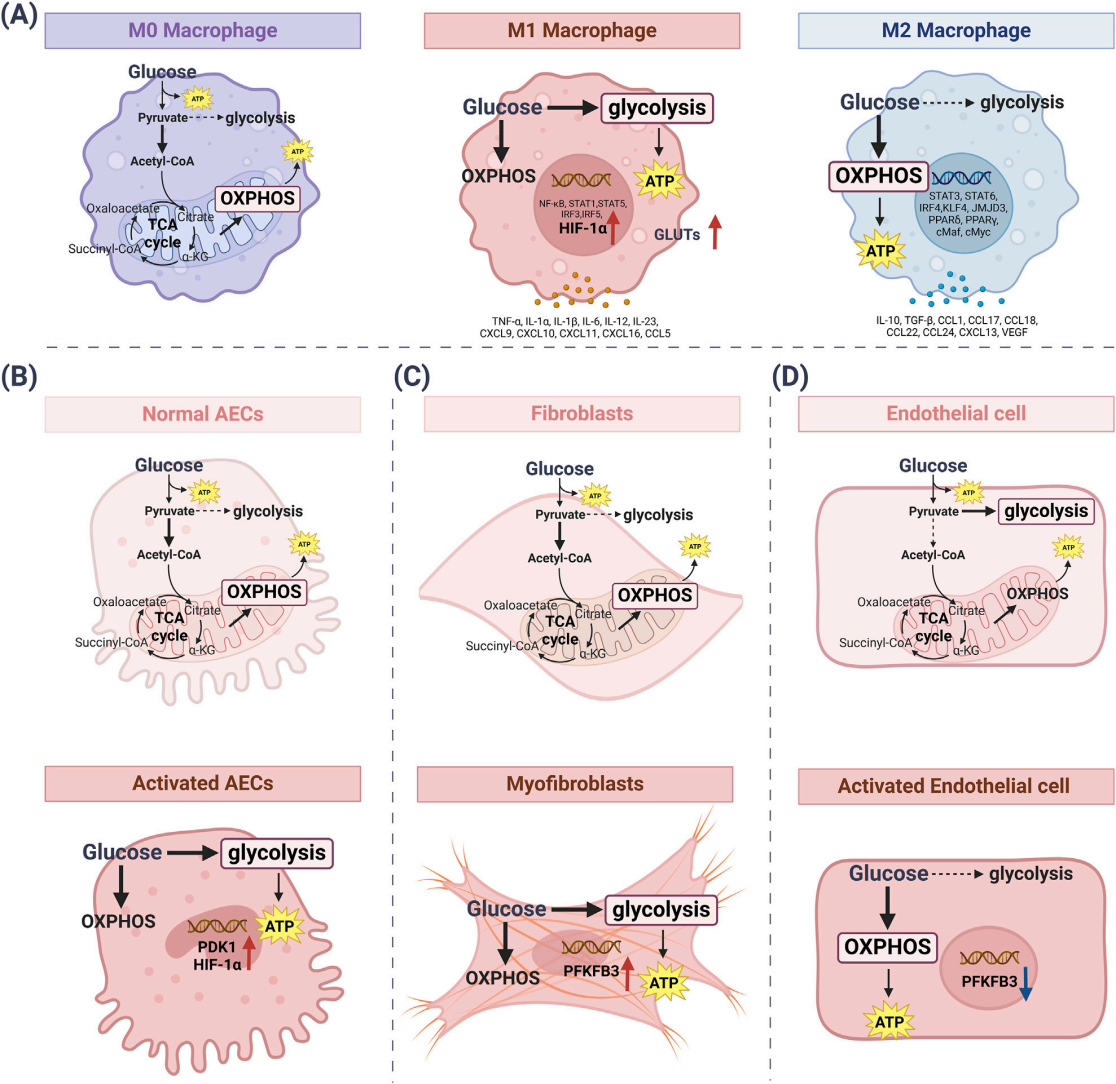
The metabolic pathways of glucose change in lung cells. **(A)** Under normal conditions, M0 macrophages obtain energy through the efficient use of OXPHOS. M1 pro-inflammatory macrophages mainly use glycolysis to synthesize ATP. Meanwhile, metabolic reprogramming activates HIF-1α and GLUTs. M2-activated macrophages use OXPHOS as a major metabolic pathway to generate ATP while increasing glucose utilization. **(B)** In normal AECs, the glycolysis rate is downregulated and OXPHOS is the main source of ATP synthesis. Activated AECs increase the rate of glycolysis, followed by increased levels of PDK1 and HIF-1α. **(C)** Fibroblasts exhibit low basal metabolism, with the main participation of the OXPHOS cycle. Myofibroblasts increase glycolysis rate, which is followed by elevated levels of PFKFB3. **(D)** Quiescent endothelial cells use glucose to produce pyruvate, which is metabolized by glycolysis. ATP synthesis also occurs via glycolysis. Activated endothelial cells mainly use OXPHOS to synthesize ATP, while PFKFB3 levels are downregulated. [ATP: adenosine triphosphate; AECs: alveolar epithelial cells; GLUTs: glucose transporters; HIF-1α: hypoxia-inducible factor-1α; OXPHOS: oxidative phosphorylation; PDK1: pyruvate dehydrogenase kinase isozyme 1; PFKFB3: 6-Phosphofructo-2-kinase/fructose-2, 6-bisphosphatase 3].

The metabolic pathways of glucose in lung cells mostly depend upon the cell types and the expression of metabolism-related enzymes in Table 2. Endothelial cells primarily rely on glycolysis to generate ATP and support vascular homeostasis. Under altered pulmonary conditions, macrophages, fibroblasts, and alveolar epithelial cells (AECs) predominantly shift toward glycolytic metabolism to meet their increased energy demands. Thus, the metabolic changes in different lung cells play an important part in lung disease (Figure 2).

**TABLE 2:** Overview of metabolic changes in various lung cells.

Cell type	Metabolic pathway	Cell state	Associated pathway	Ref.
Macrophages	OXPHOS to glycolysis	M1 macrophages	TREM-1 increases macrophage glycolysis by promoting HIF-1α expression Carbon black ultrafine activates HIF-1α and increases macrophage glycolysis, leading to emphysema	Zhong et al. (2023), Chang et al. (2022)
	Inhibited glycolysis	M2 macrophages	Increased glycolysis inhibits PF by suppressing M2 macrophage production in fibrotic mice Inhibition of the PKM2/HIF-1 pathway can reduce glycolysis and promote M1 macrophages to turn into M2 macrophages, thus weakening the acute lung injury induced by sepsis	Wang et al. (2023c), Zhang et al. (2022b)
AECs	OXPHOS to glycolysis	Activated AECs	Upregulated glycolysis is essential to support the energy expenditure that is required for cell regeneration during AECs differentiation Inhibition of HIF-1α leads to increased glycolytic activity in AECs, thereby protecting them from ARDS	Wang et al. (2023d), Roy et al. (2023)
Fibroblasts	OXPHOS to glycolysis	Myofibroblasts	In LPS-induced sepsis, increased glycolysis promoted collagen synthesis in lung fibroblasts to facilitate PF Fibroblast activation is regulated by GLUT1-dependent glycolysis in age-related lung fibrogenesis	Hu et al. (2020a), Cho et al. (2017)
Endothelial cell	Glycolysis to OXPHOS	Activated Endothelial cell	In BLM-induced pulmonary fibrosis, KD025 promoted OXPHOS and strengthened pulmonary barrier integrity in endothelial cells Shear stress activates mitochondrial OXPHOS by reducing plasma membrane cholesterol in vascular endothelial cells	Lee et al. (2020), Yamamoto et al. (2020)

### 3.1 Macrophages

Macrophages are highly heterogeneous immune cell populations. The sustained activation of macrophages further promotes the recruitment of immune cells, thereby perpetuating lung tissue inflammation, promoting tissue remodeling and fibrosis. In response to environmental stimuli, macrophages are known to exhibit plasticity by modifying and specializing their properties. Generally, macrophages can play a pro-inflammatory (M1) or anti-inflammatory (M2) role. The pro-inflammatory M1 macrophages use glycolysis as their main tool to accelerate the disease process. As expected, TREM-1 activation-induced glycolysis in M1 macrophages by facilitating HIF-1α accumulation during ARDS (Zhong et al., 2023). Chronic exposure to airborne carbon black ultrafine also activated the HIF-1α axis, which increased glycolysis in M1 macrophages, leading to emphysema (Chang et al., 2022). In COVID-19-induced severe lung injury, macrophage glucose metabolism switched to glycolysis, indirectly facilitating SARS-CoV-2 replication (Codo et al., 2020). In addition, transient receptor potential vanilloid 4 upregulated GLUT1 expression to regulate glycolysis in a stiffness-dependent manner by increasing macrophage glucose uptake during sepsis-induced lung injury (Orsini et al., 2024). Following activation, macrophages transition from oxidative phosphorylation to glycolysis, thereby enhancing their effector functions in inflammatory responses in the lung.

It is still unclear how glycolysis is involved in the function of M2 macrophages. Reports have shown that glycolysis is downregulated in M2 macrophages, affecting their polarization and biological activity (Dang and Leelahavanichkul, 2020). Macrophages from late-stage PF predominantly exhibited the M2 phenotype with reduced glycolysis. Whereas upregulation of glycolysis can suppress M2 macrophage generation in fibrotic mice, resulting in inhibition of the process of PF (Wang H. et al., 2023). Moreover, suppression of glycolysis-dependent M1 polarization via the pyruvate kinase M2 (PKM2)/HIF-1ɑ pathway switched to facilitated M2 polarization in macrophages and attenuated sepsis-induced acute lung injury (Zhang X. et al., 2022). Altogether, these data demonstrate that glucose metabolism reprogramming is a more flexible way to alter the function of macrophages.

### 3.2 AECs

AECs play a critical role in the exchange of oxygen and carbon dioxide between blood and air in the lungs, and their injury is a key process in lung disease. Glycolysis and pentose phosphate pathways are essential for AECs activation, proliferation, and regeneration. During sepsis-related ARDS, lactate levels in AECs are elevated due to the Warburg effect, which then increases glycolytic flux, promoting inflammation (Gong et al., 2017). Recent studies have shown that the expression of key glycolytic enzymes, such as pyruvate dehydrogenase kinase isozyme 1 (PDK1) is elevated in PF. Moreover, PDK1 stimulated the EMT of AECs to promote PF (Sun et al., 2024). However, upregulated glycolysis is essential to support the energy expenditure that is required for cell regeneration during AECs differentiation (Wang Z. et al., 2023). Meanwhile, inhibition of activated HIF-1α led to increased cellular glycolytic activity, which protected AECs from ARDS (Roy et al., 2023). It is known that this is the result of the interaction between AECs and AMs during ARDS. The end product of glycolysis, lactate, produced by AECs, shifts AMs towards an anti-inflammatory phenotype, thereby alleviating ARDS (Roy et al., 2023). Thus, glycolysis in AECs of lung disease provides a basis for a better understanding of lung homeostasis and injury repair.

### 3.3 Fibroblasts

Fibroblasts are the main functional cells that are activated to become myofibroblasts involved in PF. Enhanced glycolysis in myofibroblasts is essential for maintaining their pro-fibrotic phenotype. Recent research has shown that lactate produced by myofibroblasts can induce histone acetylation in the promoters of profibrotic genes in macrophages to aggravate PF (Cui et al., 2021). In LPS-induced sepsis, increased glycolysis promoted collagen synthesis in lung fibroblasts to facilitate PF (Hu X. et al., 2020). Notably, metformin has been shown to inhibit PFKFB3-associated glycolysis, thereby reducing collagen production and attenuating LPS-induced PF (Tang et al., 2021). Radiation-induced pulmonary fibrosis is a common complication of thoracic radiotherapy. It is related to the regulation of glycolysis in fibroblasts (Luo et al., 2021). Fibroblast activation is also regulated by GLUT1-dependent glycolysis in age-related lung fibrogenesis (Cho et al., 2017). Targeting glycolytic reprogramming in myofibroblasts has emerged as a promising therapeutic strategy for pulmonary fibrosis (Lai et al., 2023; Chen et al., 2021; Wang Z. et al., 2021).

Glycolysis serves as a primary metabolic pathway for rapid energy supply under adverse conditions. Cancer-associated fibroblasts (CAFs) are one of the major components of the tumor microenvironment. The study counted tumor biopsy data from LUAD patients and found a positive correlation between the degree of fibrosis and the rate of glycolytic transition in patients (Cruz-Bermudez et al., 2019). Another study reported that CAFs-secreted exosomes promoted migration, invasion, and glycolysis in hepatocellular carcinoma cells (Lu et al., 2022). Conversely, another study found that normal fibroblasts treated with TGF-β1 acquire a CAF-like phenotype characterized by upregulated glucose uptake-related genes, with minimal changes in glycolysis-driving genes (Alzaydi et al., 2023). Collectively, these findings suggest that targeting glucose metabolism reprogramming in CAFs may offer a potential adjuvant approach for cancer therapy.

### 3.4 Endothelial cells

Pulmonary microvascular endothelial cells are essential for maintaining the structural and functional integrity of the pulmonary gas exchange interface. These cells exhibit an elevated glycolytic rate and excessive endothelial proliferation, which contributes to the development of pulmonary arterial vasculopathy. In the context of pulmonary arterial hypertension, overexpression of clathrin-independent carrier proteins promotes a metabolic shift toward glycolysis, thereby enhancing proliferation in human pulmonary artery endothelial cells—a phenomenon consistent with the metabolic alterations observed in patient-derived cells (Alzaydi et al., 2023). Lung endothelial cells utilize fructose metabolism under pathological conditions. Pneumonia is associated with increased fructose levels in bronchoalveolar lavage (BAL) fluid among mechanically ventilated intensive care unit (ICU) patients. PFKFB3, a key glycolytic enzyme, has been shown in recent study to inhibit fructose-mediated glycolysis and thereby attenuate the progression of pneumonia (Lee et al., 2023). The above research demonstrates that endothelial cell hyper glycolysis, which drives vascular remodeling, represents a critical therapeutic target in the pathogenesis of pulmonary diseases. Furthermore, the dysregulation of glycolysis and OXPHOS in endothelial cells contributes to the pathological progression of pulmonary diseases. In BLM-induced pulmonary fibrosis, KD025 promoted OXPHOS and strengthened pulmonary barrier integrity in endothelial cells (Lee et al., 2020). Shear stress activates mitochondrial OXPHOS by reducing plasma membrane cholesterol in vascular endothelial cells, potentially modulating downstream signaling pathways in diseases (Yamamoto et al., 2020). Targeting endothelial cell glucose adaptation may be a potential therapeutic intervention to treat pulmonary disease.

## 4 The role of key glycolytic molecules in lung diseases

**FIGURE 3:**
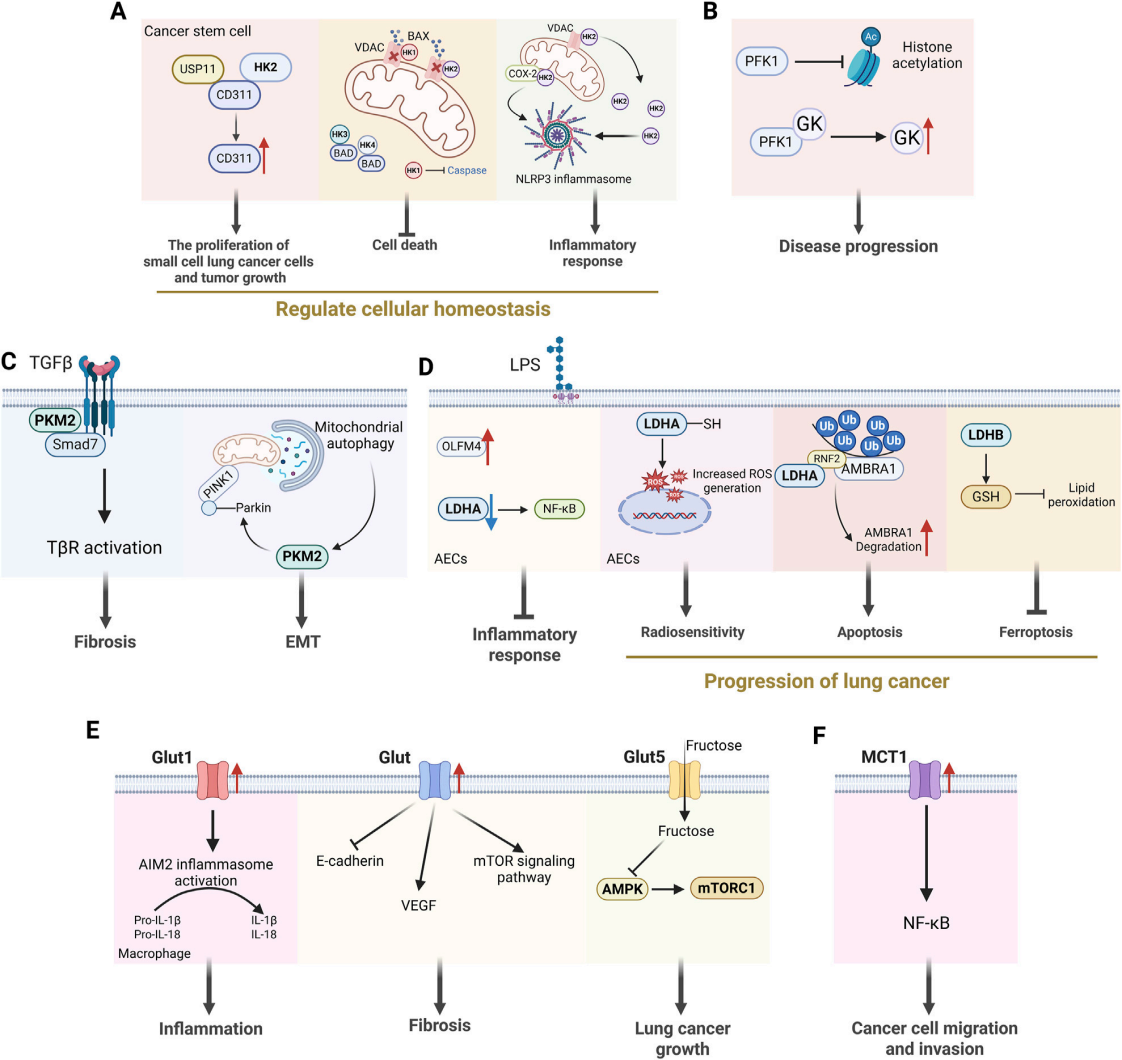
The non-glycolytic effects of key enzymes in glycolysis. **(A)** HK2 regulates cellular homeostasis by influencing cancer cell proliferation, cell death and inflammatory responses. **(B)** PFK1 can influence disease progression by affecting histone acetylation and enhancing GK activity. **(C)** PKM2 dimer can act as a direct regulator of inflammatory responses and participate in the processes of fibrosis and EMT. **(D)** LDHA is involved in inflammatory responses and the occurrence and development of lung cancer. **(E)** Glut is engaged in the development of lung diseases by influencing inflammatory responses, the occurrence of pulmonary fibrosis and lung cancer. **(F)** MCT1 activates the transcription factor NF-κB, thereby promoting the migration and invasion of cancer cells independently of its activity as a transporter. [HK2: hexokinase 2; VDAC: voltage-dependent anion channel; PFK1: phosphofructokinase-1; GK: glucokinase; PKM2: pyruvate kinase M2; EMT: epithelial-mesenchymal transition; LDHA: lactate dehydrogenase A; Glut: glucose transporter; VEGF: voltage-dependent anion channel; MCT1: monocarboxylate transporter 1].

**FIGURE 4:**
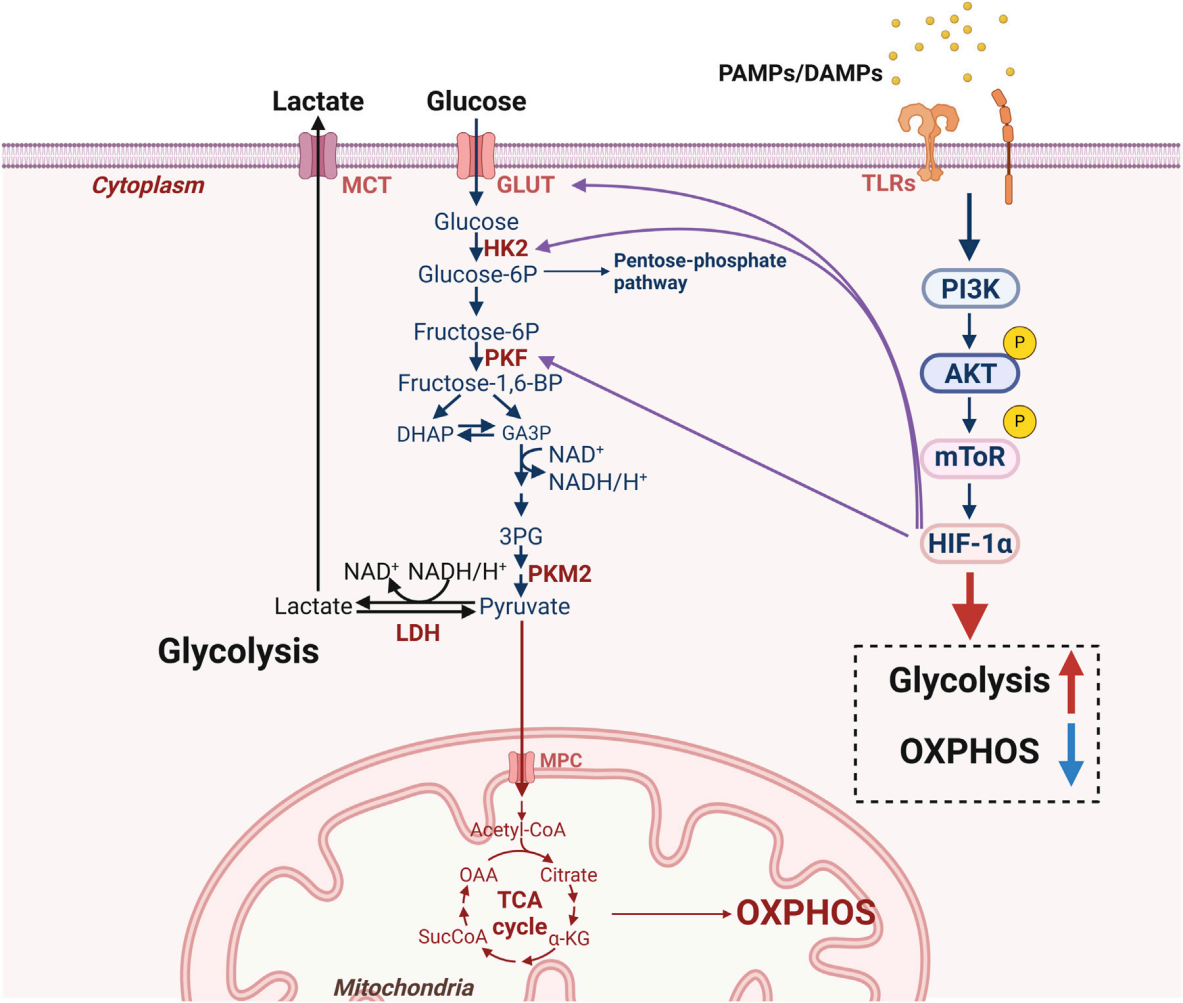
Molecular mechanisms for glucometabolic reprogramming in lung cells. Under disease conditions, DAMPs produced by damaged cells and exogenous PAMPs can activate TLRs in lung cells and then activate the AKT-mTOR pathway. Meanwhile, upregulation of HIF-1α, GLUT1 and glycolysis-related enzymes (HK2, PKF) can exacerbate glycolysis to produce lactate while inhibiting OXPHOS. [AKT: protein kinase B; DAMPs: damage associated molecular patterns; HIF-1α: hypoxia-inducible factor-1α; HK2: hexokinase 2; GLUT1: glucose transporter 1; mTOR: mammalian target of the rapamycin; MCT: monocarboxylate transporters; MPC: mitochondrial pyruvate carrier; LDH: lactate dehydrogenase; OXPHOS: oxidative phosphorylation; PAMPs: pathogen-associated molecular patterns; PKF: phosphofructokinase; PKM2: pyruvate kinase M2; TLRs: toll-like receptors].

Most studies have demonstrated that altered glucometabolic enzymes and its associated molecules play a crucial role in lung disease. Interestingly, in addition to their own function in this context, key glycolytic enzymes and molecules may also act as signaling molecules that influence the disease process (Figure 3). Therefore, understanding how glycolytic enzymes and molecules influence disease processes could pave the way for novel therapeutic approaches to treating certain lung diseases. The following sections outline the glycolytic enzymes and molecules that regulate altered glucose metabolism in lung cells during disease, and describe their respective mechanisms of action (Figure 4).

### 4.1 HK2

Hexokinase not only governs glucose metabolism to fulfill anabolic demands but also wields a pivotal influence in a wide array of cellular activities (De Jesus et al., 2022). There are five isoforms of mammalian HK: HK1, HK2, HK3, HK4 (also known as glucokinase), and hexokinase domain-containing protein 1 (De Jesus et al., 2022). HK2 is a key metabolic enzyme that catalyzes the first step of the glycolytic pathway, phosphorylates glucose to glucose 6-phosphate, and is localized predominantly in the outer mitochondrial membrane (Rho et al., 2023). HK2 is upregulated in many tumors and is an important regulator of the Warburg effect (Li et al., 2022). The study found that PD-L1 enhanced glycolysis in NSCLC by upregulating HK2, which in turn may have a dampening effect on anti-tumor immunity (Kim et al., 2019). BarH-like homeobox 2 promotes glycolysis and accelerates the process of LUAD by upregulating HK2 expression (Xie et al., 2022). These immunosuppressive effects contribute to tumor immune evasion. It has been demonstrated that the targeted degradation of HK2, the mediator of cellular glycolysis, can inhibit the aggregation of immune cells in the lung and attenuate acute lung injury (Yang et al., 2024).

In addition, the non-metabolic function of HK2 plays a significant role in the development of lung disease. Non-mitochondrial HK2 directly interacted with CD133, which inhibited CD133 polyubiquitylation and degradation, thereby promoting small cell lung cancer cell proliferation and tumor growth (Wang et al., 2022). The binding of HK1 and HK2 to the voltage-dependent anion channel at the mitochondrial outer membrane restricts access to the proapoptotic BCL-2 family member BAX and prevents apoptosis induction (Ciscato et al., 2020; Arzoine et al., 2009). HK3 and HK4 has also been found to play a role in balancing apoptotic activity as it can interact with proapoptotic BCL-2 family member BAD, whose protect the cells from death (Danial et al., 2003; Seiler et al., 2022). In addition, HK1 has been demonstrated to inhibit the formation of active, pro-apoptotic caspases in response to extrinsic inducers of apoptosis (Schindler and Foley, 2010). The relationship between this process and the regulation of cell death is a fascinating area of research, particularly given the association of inflammasome activation with pyroptosis. The study established that dissociation of HK from mitochondria was sufficient to trigger NLRP3 inflammasome assembly and activation in response to bacterial infection (Wolf et al., 2016). Interestingly, the release of HK2 from the mitochondria has been shown to result in the formation of VDAC oligomers, which in turn recruit NLRP3, thereby initiating inflammasome assembly in LPS-primed macrophages (Baik et al., 2023). Palmitoylation of COX-2Cys555 enhanced its interaction with HK2 to regulate NLRP3 inflammasome activation and pyroptosis in COVID-19 syndrome (Wu et al., 2025). Consequently, HK appears to be poised to influence this balance of multiple types of cell death and the resultant inflammation. From this, we can see that HK effectively integrates multiple responses such as energy production, cell survival pathways, mitochondrial homeostasis maintenance, and immune responses to achieve regulation of cellular homeostasis.

2-Deoxy-D-glucose (2-DG), a glucose analog, acts as a competitive inhibitor of glucose metabolism by targeting HK, thereby suppressing glycolysis at this rate-limiting step (Ruan et al., 2017). Significant advancements have been made in research on 2-DG. Studies demonstrate that 2-DG effectively blocks glycolysis, ameliorates systemic inflammation in mice with polymicrobial sepsis, and mitigates sepsis-associated lung injury (Payen et al., 2017). In cancer cells, 2-DG is phosphorylated by HK into 2-deoxy-D-glucose-6-phosphate (2-DG-6P), which cannot proceed through subsequent glycolytic steps. The accumulation of 2-DG or 2-DG-6P leads to ATP depletion, cell cycle arrest, and cancer cell death. Research indicates that 2-DG significantly reduces the size of tumor spheroids in mouse lung adenocarcinoma cells (Certo et al., 2021). Furthermore, co-delivery systems (e.g., the dual-drug delivery system 2-DG + α-TOS @FR) enhance drug targeting and therapeutic efficacy (De Backer et al., 1997).

### 4.2 Phosphofructokinase-1 (PFK1)

PFK1 is a central enzyme in the third step of the glycolytic pathway, catalyzing the production of fructose-6-phosphate to fructose-1,6-bisphosphate, a product that positively activates PFK-1 and promotes sugar catabolism (Chen et al., 2023; Mor et al., 2011). PFK-1 is the least catalytically efficient of the key glycolytic enzymes and is therefore the rate-limiting enzyme of glycolysis (Zuo et al., 2021). The rate of glycolysis is strictly dependent on the activity level of the enzyme. PFK-1 consists of four subunits and is a tetramer (Webb et al., 2017). Fascin promotes the growth and metastasis of lung cancer cells by increasing glycolysis through increased expression and activity of PFK1 and PFK2 (Lin et al., 2021). In contrast, the citrate strategy can effectively suppress PFK1/PFK2 expression and reverse dedifferentiation in a RAS-driven lung cancer model (Icard et al., 2022).

Although the non-metabolic effects of PKF1 in lung disease remain unclear, these effects have been investigated in other pathological conditions. A recent study has indicated that PFK1 can play a role in disease progression by influencing post-translational modifications of proteins (Wang R. et al., 2024). In addition, PFK1 enhances GK activity through direct binding (Massa et al., 2004). Taken together, PFK1 plays an important role in the regulation of glycolysis in lung cancer, and its non-metabolic effects need to be further investigated.

### 4.3 PKM2

PKM converts phosphoenolpyruvate to pyruvate, a metabolite of glycolysis, and is a key enzyme in the regulation of glycolysis (Massa et al., 2004). PKM1 and PKM2 are alternatively spliced isoforms of the PK enzyme. They are very strongly associated with the progression of lung diseases. PKM1 promotes malignancy in small-cell lung cancer by activating glucose catabolism (Morita et al., 2018). Hypoxia-induced production of lncRNA-AC020978 enhances the stability of PKM2 protein and promotes the proliferation of NSCLC through direct interaction with PKM2 32308748 (Morita et al., 2018). PKM2 S287 phosphorylation and tetramer formation enhance OXPHOS, thereby enhancing cancer cell survival and proliferation (Comandatore et al., 2022). Enhanced PKM2 activity also promotes glycolysis in LUAD cells and promotes tumor progression (Rogatzki et al., 2015; Yin et al., 2020; Wang L. et al., 2024; Deng et al., 2022). Quercetin protects mice from glycolysis-induced lung injury by suppressing PKM2 nuclear accumulation through SIRT1 (Feng Y. et al., 2024). Activation of Sirtuin 3 (SIRT3) by procyanidin B2 leads to K433 deacetylation of PKM2 to reduce glycolysis, which alleviates lung ischemia/reperfusion injury (Wang Q. et al., 2025). The expression levels of PKM2 can change the activation of lung cells and their metabolic state.

PKM2 can be either a tetramer, which has a similar function to PKM1, or a dimer, which loses its glycolytic activity but performs other non-glycolytic functions. PKM2 promotes the progression of fibrosis by directly interacting with Smad7 and enhancing TGF-β1 signaling (Gong et al., 2021). The protein PKM2 controls the inflammation of the airways and the process of EMT (epithelial-to-mesenchymal transition) caused by cigarette smoke by influencing the PINK1/Parkin-mediated mitophagy (Zhao et al., 2025). Therefore, the PKM2 dimer appears to fulfil a distinct function as a direct regulator of inflammatory programmer. Small molecule inhibitor PKM2-in-1 (compound 3k) can reduce breast cancer drug resistance by inhibiting the expression of PKM2 (Zhang H. et al., 2022). In recent years, studies have shown that Vitamin K5 can also specifically inhibit PKM2, but its inhibitory effect on PKM1 and PKL is weak, and it is mostly used in tumor research (Jansen et al., 2009).

### 4.4 Lactate dehydrogenase (LDH)

LDH is a class of NAD-dependent kinases that exists in three subunits, LDHA, LDHB, and LDHC, and can constitute six tetrameric isozymes (Comandatore et al., 2022). This enzyme is essential in the glycolytic pathway, catalyzing the reversible conversion of pyruvate to lactate, as well as the conversion of nicotinamide adenine dinucleotide (NADH) to NAD^+^ to maintain glycolytic flux (Rogatzki et al., 2015). LDHA-driven lactate efflux facilitates multiple oncogenic processes. It has been shown that upregulation of LDHA expression promotes the proliferation and migration of lung cancer cells and reduces the survival rate of lung cancer patients (Yu et al., 2018; Comandatore et al., 2022; Yin et al., 2020; Wang L. et al., 2024). LDHA is predominantly responsible for the conversion of pyruvate to lactate, thereby supporting glycolysis under both anaerobic and aerobic conditions. In contrast, LDHB exhibits a higher affinity for lactate, catalyzing its conversion back to pyruvate, which in turn fuels OXPHOS by linking it to the TCA cycle. Silencing of LDHB induces sustained mitochondrial DNA damage, and reduces mitochondrial respiratory complex activity and OXPHOS, resulting in reduced levels of mitochondria-dependent metabolites (e.g., TCA intermediates, amino acids, and nucleotides), which inhibits NSCLC development (Deng et al., 2022).

In addition to its capacity to directly modulate the metabolic function of the cancer cell, there is evidence to suggest that LDHA may also be capable of directly influencing inflammatory responses. The increased levels of LDHA phosphorylation and the downstream NF-κB activation induced by LPS in epithelial cells were effectively diminished by OLFM4 overexpression and recombinant OLFM4 treatment, thereby reducing LPS-induced pro-inflammatory responses in lung epithelial cells (Gong et al., 2021). Furthermore, elevated LDHA expression in tumor cells was found to be associated with activation of the NF-κB pathway in LUAD (Wang G. et al., 2024). Protein posttranslational modification has the capacity to alter the activity of LDHA, in turn affecting downstream effector molecules. The loss of S-nitrosylation in LDHA after irradiation increased radiosensitivity by generating ROS in human pulmonary epithelial cells (Feng Y. et al., 2024). LDHA modulates the cell cycle and apoptosis by promoting the ubiquitination and subsequent degradation of AMBRA1, thus inducing cisplatin resistance in LUAD (Wang Q. et al., 2025). The suppression of LDHB has been shown to reduce SLC7A11-dependent glutathione metabolism, thereby protecting KRAS-mutant NSCLC from ferroptosis (Zhao et al., 2025). The relationship between LDH non-metabolic function and the level of flux through the glycolytic cascade or enzymatic activity in lung diseases remains to be elucidated.

Inhibitors of LDH are mainly pyruvate analogs or NADH analogs, which inhibit cellular energy metabolism through competitive inhibition (Zhang et al., 2019). Oxalic acid acts as a specific inhibitor of LDH and is a structural pyruvate analog (Chen Y. et al., 2024). Studies have shown that oxalic acid inhibits the conversion of pyruvate to lactate by inhibiting LDH and glycolysis, thus alleviating acute lung injury induced by sepsis (Payen et al., 2017). At the same time, Oxalic acid combined with paclitaxel has a good synergistic inhibitory effect on tumor cells. FX-11 is a selective reversible inhibitor of LDH that competes with NADH and shows significant antitumor activity in xenografts of lymphoma and pancreatic cancer (Gu et al., 2022; Yang K. et al., 2022). It is worth noting that the research of glycolysis inhibitors has shifted from single-target inhibition to multi-mechanism collaborative intervention, and combined with emerging nano delivery technologies, is expected to break the existing treatment bottleneck. It is believed that soon, the combination of glycolysis inhibitors with other therapeutic agents can play a greater therapeutic role in specific drug delivery systems.

### 4.5 Glucose transporters (GLUTs)

The GLUT transporter protein, a member of the SLC2A gene family, facilitates glucose transport across the mammalian plasma membrane (Holman, 2020). There are 14 known isoforms of the human SLC2A gene family, which encode different GLUT proteins (Thorens and Mueckler, 2010). The GLUT proteins have different specificities for substrates, transport kinetics, and tissue expression patterns (Mueckler and Thorens, 2013).

GLUT1 is the most important and widely expressed isoform of the GLUT family of factors (Cornwell et al., 2023). GLUT1 plays a pivotal role in fundamental glucose uptake (Veys et al., 2020). The GLUT1 protein is present in fetal lung tissue, but its expression decreases as the lungs develop (Macheda et al., 2002). However, GLUT1 is also commonly found to be highly expressed in lung cancer cells in the context of disease (Ancey et al., 2021). Increased GLUT1 expression in NSCLC reduces tumor cell differentiation and increases cell proliferation by mediating cellular glycolysis (Xu et al., 2022; Kokeza et al., 2023). Furthermore, high GLUT1 expression enhances macrophage immune response by affecting glucose metabolism, which in turn exacerbates ARDS (Deng et al., 2020). GLUT3 is expressed in olfactory epithelial cells, and its expression is particularly elevated in lung tumor cells undergoing an epithelial-mesenchymal transition (Pantaleon et al., 1997; Masin et al., 2014). Concurrently, GLUT3 expression has been observed to be associated with increased immune cell infiltration in the tumor microenvironment. In the context of LUSC, the glucose uptake capacity of cancer cells is comparatively diminished when GLUT3-mediated glucose uptake by immune cells is augmented in tumors. The GLUT-ratio could be a useful tool for evaluating differential glycolysis activation in cancer and cancer-infiltrating immune cells (Na et al., 2020). The preceding studies indicate that GLUTs modify cell fate by impacting glycolytic processes.

However, recent research is beginning to reveal the potential for GLUTs to function in a non-metabolic role. GLUT1 has been shown to activate AIM2 inflammasome in a dose-dependent manner during the process of fibrosis exacerbation (Cho et al., 2020). Increased GLUT expression has been demonstrated to directly inhibit the expression of E-cadherin in bronchial cells in asthma (Lv et al., 2024), promote the expression of vascular endothelial growth factor A in epithelial cells in ARDS (Liang et al., 2024), and activate the mTOR signaling pathway to exacerbate lung fibrosis (Andrianifahanana et al., 2016). Furthermore, GLUT5-mediated fructose utilization has been demonstrated to activate mTORC1 activity, thereby promoting lung cancer growth (Chen et al., 2020). GLUTs have been a therapeutic target for many solid tumors, and elucidating the role of GLUT in lung disease offers the potential to develop a cure.

### 4.6 Monocarboxylate transporter (MCT)

Monocarboxylic acids such as lactate, pyruvate, and ketone bodies are key substances involved in glycolysis (Pierre and Pellerin, 2005; Pellerin et al., 2005). Cells require precise regulation of these substances to survive and grow properly. A variety of proteins that transport monocarboxylic acids exist in cells, the most prominent of which is MCT, which belongs to the Solute Carrier family (Wang N. et al., 2021; Zhang et al., 2020). In general, MCT1 is involved in the uptake or efflux of lactate, while MCT4 is mainly involved in the entry of lactate from glycolytic cells into the microenvironment (Kobayashi et al., 2021). The EMT process in AECs is thought to be associated with the development of pulmonary fibrosis. It has been shown that LPS inhibits MCT1 expression in mouse AECs, inducing impaired lactate transport and leading to lactate accumulation, which eventually promotes the process of EMT and lung fibrosis (Feng J. et al., 2024). Reduced MCT1/4 expression in distal airway epithelium may disrupt lung branching morphogenesis, thereby promoting the development of lung hypoplasia in a nitrogen-induced congenital diaphragmatic hernia model (Takahashi et al., 2016). Upregulation of MCT4 expression increases lactate content in the microenvironment of NSCLC and further promotes cancer cell proliferation, migration, and angiogenesis (Markou et al., 2021; Lee et al., 2011; Ruan et al., 2017). MCT plays an important role in the glycolytic pathway due to its transport capacity, making it a potential target for lung disease therapy. Interestingly, recent research has shown that MCT1 activates the transcription factor NF-κB, thereby promoting cancer cells migration and invasion independently of its activity as a transporter (Payen et al., 2017). Consequently, the function of MCT in lung diseases, beyond its role as a transporter, requires further investigation.

## 5 The role of glucose metabolites in lung diseases

Glucometabolic reprogramming in disease is characterized by alterations in metabolic pathways and the accumulation of metabolites from glucose metabolism. These metabolites often function as signaling molecules that influence important biological processes such as cell activation, proliferation and differentiation. Another consequence of metabolite accumulation may involve the post-translational modification, which has the potential to affect the function of the key protein in lung diseases. Therefore, summarizing the specific roles of glucose metabolites in disease may provide new ideas for disease treatment (Table 3).

**TABLE 3:** The role of glucose metabolites in lung diseases.

Glucose metabolite type	Content change	Associated pathway	Post-translational modifications	References
Lactate	Increase	Activate the signaling pathways that regulate inflammation: activates macrophages to differentiate into a pro-fibrotic phenotype, secreting factors like TGF-β to drive pulmonary fibrosis	Lactylation: (1) lactylation of histone H3K18 (2) lactylation of meiotic recombination 11 and HMGB1	Cui et al. (2021), De Backer et al. (1997), Wang et al. (2024e), Zhang et al. (2019)
		Activate the tumor immune tolerance cell signaling pathway: Lactate acidifies the tumor microenvironment, promotes the invasion and metastasis of cancer cells, and simultaneously inhibits the function of T cells to facilitate immune escape		Gu et al. (2024), Zhang et al. (2024b)
Citrate	Increase	Facilitate the proliferation of cancer cells: In tumor metabolic reprogramming, the accumulation of citrate may partially inhibit glycolysis while paradoxically promoting adipogenesis to support the proliferation requirements of cancer cells	Acetylation modification: (1) acetylation of H3 histone (2) acetylation of ACLY by PCAF/SIRT2 and STAT3	Li et al. (2023), Govender et al. (1998)
		Damage lung parenchymal cells and lung immune cells: (1) As DAMPs to activate macrophages, promoting the lung injury induced by LPS. (2) Binding to the mitochondrial protein FUNDC1 triggers aberrant autophagy and necroptosis that compromise alveolar epithelial integrity. (3) Redirect metabolic fluxes to other pathways to regulate immune cell function and disease progression		Duan et al. (2021), Yang et al. (2022c), An et al. (2024)
Succinate	Increase	Proliferation of cancer cells: (1) Causing macrophages to become tumor-associated macrophages	Succinylation: succinylation of succinyl-coenzyme A (CoA) synthetase GDP-forming subunit β, succinylation of GAPDH, succinylation of SOD2	Yuan et al. (2025), Wu et al. (2020)
		Immune-inflammatory responses: (1) The supplementation of exogenous succinates leads to a significant increase in inflammatory responses, thereby aggravating ARDS. (2) Activate HIF-1α-mediated inflammation in lung contusion. (3) Enhanced mitochondrial oxidative stress promotes cell apoptosis in the process of lung ischemia-reperfusion injury		Mills et al. (2016), Wang et al. (2023e) Yu et al. (2023), Liu et al. (2024), Wang et al. (2025b), Hu et al. (2023)

### 5.1 Lactate

Lactate is a key metabolic byproduct from cells that use glycolysis for energy. It is a strong acid with a pKa value of 3.86, and it influences tissue pH (Certo et al., 2021). Lactate levels significantly increase in both lung tissue and serum, indicating a critical role of lactate in either exacerbating or ameliorating lung diseases outcomes (De Backer et al., 1997). Lactate has been found to initiate cell signaling pathways that regulate inflammatory progression and tumor immune tolerance. In hypoxic lung diseases (e.g., pneumonia, COPD, ARDS), tissue hypoxia activates glycolysis, leading to lactate accumulation, with its levels positively correlating with disease severity (Wang HX. et al., 2024). In the tumor microenvironment, cancer cells continuously produce lactate via the Warburg effect, where lactate not only acidifies the microenvironment to promote invasion and metastasis but also facilitates immune escape by suppressing T-cell function (Gu et al., 2024; Zhang C. et al., 2024). Additionally, lactate activates macrophages to differentiate into a pro-fibrotic phenotype, secreting factors like TGF-β to drive pulmonary fibrosis (Cui et al., 2021). Clinically, blood lactate monitoring serves as a key indicator for assessing tissue perfusion and prognosis, while drugs targeting LDHA or lactate transporters show therapeutic potential in animal models (Zhang H. et al., 2022; Jansen et al., 2009; Zhang F. et al., 2024). These findings suggest that modulating lactate metabolism may offer novel strategies for precise treatment of pulmonary diseases.

Lactylation, a post-translational modification first identified in 2019, involves the utilization of lactate, as a substrate for histone lactylation (Zhang et al., 2019). This process has been demonstrated to exert a direct influence on the transcription of chromatin-associated genes. The study found that lactate accumulation induced M2 macrophage polarization, impaired CD8^+^ T cell function, and upregulated immunosuppressive genes in LUAD. Furthermore, histone H3K18 lactylation in macrophages has been demonstrated to exacerbate this immunosuppressive state (Wu et al., 2025). Interestingly, non-histones have also been found to undergo lactylation. Non-histone lactylation exhibits broader functional impacts. In DNA repair, lactylation of meiotic recombination 11 enhances DNA-binding capacity and promotes homologous recombination repair, leading to chemotherapy resistance (Chen Y. et al., 2024). For immunomodulation, lactylation suppresses the cytotoxic function of CD8^+^ T cells while promoting the immunosuppressive activity of Tregs, thereby shaping an immunosuppressive tumor microenvironment (Gu et al., 2022). Further research demonstrates that, in Sepsis, the lactylation of HMGB1 (an inflammatory factor) by macrophages is facilitated through a P300/CBP-dependent pathway, thereby compromising the integrity of endothelial cells (Yang K. et al., 2022). Overall, lactylation can ultimately increase the susceptibility to diseases by regulating the expression of genes related to the inflammatory response and cell polarization. Nevertheless, further investigation is required into the complex regulatory networks of lactylation and its cell-type-specific effects.

### 5.2 Citrate

As a core intermediate of the TCA cycle, citrate plays a critical role in the regulation of glucose metabolism (Parkinson et al., 2021). Citrate effectively reduces the rate of glucose breakdown by allosterically inhibiting PFK-1, the rate-limiting enzyme of glycolysis (Parkinson et al., 2021). This inhibition prevents futile glucose breakdown during energy surplus. Concurrently, citrate serves as the product of pyruvate conversion entering the TCA cycle, providing cellular energy and participating in biosynthetic processes such as fatty acid synthesis. In the context of tumor metabolic reprogramming, the accumulation of citrate exhibits a dual function. It may partially suppress glycolysis while paradoxically promoting lipogenesis to support the proliferation demands of cancer cells (Lin et al., 2013). Citrate accumulation also plays a complex dual role by intertwining metabolic dysregulation with damage to immune cells. As a pivotal TCA cycle intermediate, citrate serves as a DAMP to activate macrophages and promote LPS-induced lung injury (Duan et al., 2021). Concurrently, excessive citrate binds to mitochondrial protein FUNDC1, triggering aberrant autophagy and necroptosis that compromise alveolar epithelial integrity, as observed in acute lung injury (Yang HH. et al., 2022). Additionally, citrate has been shown to redirect metabolic flux towards alternative pathways (e.g., itaconate biosynthesis), thereby further modulating immune cell function and disease progression (An et al., 2024). The findings emphasize the effects of citrate, insofar as while its metabolic by-products fuel pathological inflammation, its accumulation directly induces cell death.

The core mechanism of citrate-mediated post-translational modification involves the catalysis of citrate by ATP-citrate lyase (ACLY), resulting in the generation of acetyl-CoA (Wellen et al., 2009). This acetyl-CoA then serves as the acetyl donor for the dynamic regulation of histone and non-histone acetylation, thereby influencing inflammation, cell death and immunometabolism (Wellen et al., 2009). Citrate accumulation has been demonstrated to promote acetyl-CoA production via the STAT3/ACLY axis, thereby driving acetylation at H3 histone in *M. pneumoniae* infection (Yang et al., 2025). It also has been shown that citrate accumulation leads to histone acetylation, which triggers chromosome structure dissolution and initiates inflammatory gene transcription in septic lung injury (Li et al., 2023). Furthermore, the acetylation of ACLY by PCAF/SIRT2 has been demonstrated to regulate its stability, creating a positive feedback loop that amplifies metabolic dysregulation in lung cancer (Lin et al., 2013). In the context of non-histone modifications, malonylation of GAPDH and acetylation of STAT3 have been observed to disrupt glycolysis and macrophage polarization, respectively (Yang et al., 2025). Conversely, HDAC10-mediated deacetylation has been shown to inhibit M2 polarization, thereby contributing to the alleviation of asthma symptoms (Zhon et al., 2023). The citrate-acetyl-CoA-acetylation axis integrates metabolic signals and epigenetic modifications to form a positive feedback loop that amplifies inflammation, promotes cell death, and disrupts immunometabolism homeostasis. Therapeutic targeting of this axis necessitates a balance between anti-inflammatory effects and pathogen clearance capacity, in order to circumvent the potential risk of immunosuppression.

### 5.3 Succinate

Succinate is generated in the mitochondrial matrix in a reversible three-step reaction catalyzed by the TCA cycle enzyme succinyl-CoA synthetase. Recent studies have demonstrated that succinate accumulation is observed in lung disease, exhibiting a significant correlation with cancer cell proliferation and immune-inflammatory responses (Mills et al., 2016; Wang YH. et al., 2023; Yuan et al., 2025; Yu et al., 2023). The exogenous supplementation of succinate resulted in a significant increase in the inflammatory response, thus exacerbating the severity of ARDS (Liu et al., 2024). Moreover, succinate can act as a signaling molecule to activate HIF-1α-mediated inflammation in lung contusion (Suresh et al., 2023). Succinate accumulation also impacts disease progression through the modulation of cellular differentiation. It has been demonstrated that cancer cells release succinate into their microenvironment, thereby activating succinate receptor signaling to polarize macrophages into tumor-associated macrophages, which is a critical step in cancer metastasis (Wu et al., 2020). During lung ischemia-reperfusion injury, succinate accumulates, thereby enhancing mitochondrial oxidative stress and promoting cell apoptosis (Wang W. et al., 2025). Another potential consequence of succinate accumulation is the occurrence of lysine succinylation, a post-translational modification. In LUAD, succinylation on Lys93 increases the succinyl-coenzyme A (CoA) synthetase GDP-forming subunit β stability, leading to metabolic reprogramming and tumor progression (Hu et al., 2023). Smoking modulates lung cancer cell metabolism through enhanced succinylation of lysine 251 on GAPDH, a key glycolytic enzyme, thereby promoting the NSCLC (Wang K. et al., 2025). Succinate can reduce the enzymatic activity of SOD2 through succinylation modification at the K68 site in allergic airway inflammation (Wang C. et al., 2025). Succinylation of proteins is a significant process in the development of metabolic changes associated with lung diseases; however, the mechanisms by which succinate accumulates in various lung cells remain to be fully elucidated.

## 6 Signaling pathways for glucose metabolism in lung diseases

Lung cells can regulate the inflammatory response in the lungs as well as the progression and resolution of the inflammatory process through glucose metabolism pathways. The intricate regulation of glucose metabolism through the inflammatory response in lung cells encompasses a myriad of interconnected signaling pathways, highlighting the complexity and vitality of this process. In the following sections, we will explore the primary signaling pathways and mechanisms that regulate glucose metabolism changes in lung cells within the inflammatory microenvironment.

### 6.1 mTOR

mTOR is a highly conserved serine/threonine protein kinase, a member of the Phosphatidylinositol-3 kinase-related kinase (PIKK) family of proteins (Mossmann et al., 2018). mTOR exists in two distinct catalytic subunit complexes, mTORC1 and mTORC2, which have different compositions and functions (Hua et al., 2019). These two mTOR complexes play very different roles in the regulation of metabolism and cell proliferation (Marques-Ramos and Cervantes, 2023). mTORC1 is a key regulator of environmental and hormonal signaling, activating anabolic processes and inhibiting catabolic processes when nutrients such as amino acids, glucose, cholesterol, and nucleotides are in abundance (Szwed et al., 2021; Xu C. et al., 2023; Hosios et al., 2022). mTORC2 is more responsive to activation of PI3K by insulin, IGF-1 or leptin (Ragupathi et al., 2024; Kma and Baruah, 2022; Mohlin et al., 2015).

Studies show that certain PAMPs, such as LPS, and DAMPs, such as ATP, activate the mTOR pathway (Hu Y. et al., 2020). TREM-1 promotes macrophage HIF-1α expression through activation of the PI3K/AKT/mTOR pathway, increases glycolysis, and activates NOD-like receptor protein 3 (NLRP3) inflammasome (Zhong et al., 2023). Inhibition of mTOR inhibited TREM-1-induced metabolic reprogramming and NLRP3/caspase-1 activation in macrophages (Zhong et al., 2023). This highlights the important role of glycolysis driven by mTOR in the activation of the inflammatory process.

The presence of LPS may trigger a process in lung fibroblasts known as aerobic glycolysis by activating the PI3K-Akt-mTOR/PFKFB3 pathway, which can be reversed by using the mTOR inhibitor rapamycin (Hu X. et al., 2020). Moreover, PTPRH can activate the PI3K/AKT/mTOR signaling pathway to promote glycolysis, proliferation, migration, and invasion of NSCLC cells, and ultimately promote tumor progression (Wang S. et al., 2023). As such, mTOR is a key target in lung diseases and an understanding of its activity and the complexes that regulate it is essential for the development of more effective therapeutic regimens.

### 6.2 HIF-1α

The Hypoxia-Inducible Factor 1 Complex (HIF-1) is now widely recognized as a major regulator of the response to hypoxia (low oxygen levels) (Wu Q. et al., 2022). HIF-1 is a heterodimeric transcription factor complex consisting of two subunits: the HIF-1α subunit, which responds to O_2_, and the HIF-1β subunit, which is structurally expressed (Wang et al., 1995). The intact HIF-1 complex binds to the Hypoxia response element (HRE) in the promoters of genes that control various processes such as metabolism, proliferation, apoptosis, and angiogenesis (Liu et al., 2018). HIF-1α, whether acting in a pro- or anti-inflammatory capacity, operates downstream of mTOR and is instrumental in overseeing the metabolic reprogramming and functional characteristics of lung cells (Konieczny et al., 2022; Cheng et al., 2014).

Under aerobic conditions, aberrant HIF-1α activity leads to a metabolic shift towards aerobic glycolysis (Reyes et al., 2020). Activation of mTOR and upregulation of HIF-1α expression when lung cells enter a pro-inflammatory state (Hu X. et al., 2020; Wu H. et al., 2022). Downregulation of SIRT3 promotes mitochondrial fission and oxidative stress in mouse lung tissue cells by increasing HIF-1α expression, leading to elevated ROS production in lung cells, thereby exacerbating lung ischemia-reperfusion injury (LIRI) (Liu et al., 2022). Furthermore, hyperactivation of HIF-1α leads to alveolar epithelial senescence and increased secretion of senescence-associated secretory phenotypes (SASPs), which ultimately exacerbates smoking-induced COPD (Wu H. et al., 2022). These findings suggest that HIF-1α regulates the cellular state of the lung through glycolysis. Notably, key genes involved in glycolysis such as GLUT1, HK2, and PFKFB3 are directly regulated by HIF-1α, highlighting the intimate connection between HIF-1α and cellular glycolysis in the lung (Ferrer et al., 2014; You et al., 2022; Shen et al., 2025).

It has been demonstrated that, remarkably, glycolysis-related enzymes not only facilitate tumor growth, but also regulate the metabolic and functional status of immune cells within the tumor microenvironment. The expression of enzymes such as HK1/2, PFK1, LDHA and GLUT1 is elevated, resulting in an increase in glycolytic flux and, consequently, excessive lactate production. The elevated levels of lactate have been shown to provide nutrients for tumor cells and concurrently acidify the microenvironment of the tumor, resulting in impaired T cell proliferation and effector function. This process has been observed to promote the differentiation of Tregs, thus contributing to immune suppression (Zhu et al., 2025). By targeting the PI3K/Akt/mTOR pathway or mTOR/HIF-1α pathway, the expression and activity of key glycolytic enzymes (e.g., HK, PFK, PK) are reduced. This process has the effect of reducing the production of lactate, while also reducing acidification of the tumor microenvironment, inhibiting Treg induction and enhancing both the proliferation and function of T cells (Zur et al., 2025). Consequently, the targeting of critical molecules involved in glucose metabolism has been demonstrated to exert a dual benefit in tumor therapy, manifesting in the impairment of tumor cell growth and the promotion of antitumor immunity.

## 7 Conclusion and perspectives

The lungs are the largest organ in the human body and the diseases affecting them are many. Currently, numerous studies have demonstrated that glucose metabolism reprogramming plays a critical role in lung diseases and is intricately linked to immune responses. The bidirectional regulatory mechanisms between glucose metabolism and immunity are primarily reflected in the activation states of immune cells. Beyond the metabolic reprogramming discussed in this review, which influences macrophage polarization and the tumor microenvironment. T cell dynamics in lung diseases, particularly cancer, are also closely associated with altered glucose metabolism (Angelin et al., 2017). T cells predominantly rely on oxidative phosphorylation as their primary energy source; however, upon activation, they switch to glycolytic metabolism via the mTOR-HIF-1α signaling axis, which supports cellular proliferation and effector functions (Zhao et al., 2021). Within the tumor microenvironment, the glycolytic metabolite lactate not only directly suppresses NK cell function but also facilitates immune evasion through epigenetic modifications (Harmon et al., 2019). However, the roles of other metabolites in immune escape remain to be fully elucidated. Research on cancer cells has uncovered multiple non-metabolic functions of glycolytic enzymes, which have only recently been explored in the context of inflammation. Just as cellular metabolic activity informs our understanding of bioenergetic requirements, the non-metabolic roles of glycolytic enzymes and their derivatives may provide novel insights into the interplay between metabolism and inflammation. Importantly, compared to therapies that profoundly alter enzymatic activities within metabolic pathways, these alternative strategies may offer more translatable therapeutic options for clinical application.

In addition, manipulating the neurometabolic reprogramming of lung cells, which in turn modulates the lung microenvironment, may become another feasible option for reversing most lung diseases. Thus, reprogramming glucose metabolism has become an emerging field with important contributions to make to understanding lung disease. In this review, we have shown that glucose metabolism reprogramming, directly or indirectly affecting lung immune and structural cells, promotes the development of diseases. Previous studies have frequently demonstrated the direct influence of sugar metabolites on inflammatory responses, including both pro-inflammatory and anti-inflammatory effects. Current research increasingly emphasizes the specific roles of sugar metabolite accumulation in disease pathogenesis, such as their modulation of signaling pathways and regulation of cell fate. Therefore, while accumulating evidence suggests potential associations, whether glucose metabolites can serve as reliable biomarkers for assessing lung disease severity remains to be further investigated. Additionally, understanding the cellular localization of metabolite accumulation and its impact on the immune microenvironment remains an area requiring further exploration. Targeting altered lung cell metabolism may be a valuable avenue for the development of therapies for lung disease. Nevertheless, significant gaps remain in our understanding of the impact of glucose metabolism on lung disease. It is well known that the lungs are one of the organs most in contact with the environment. With the development of chemical science and technology, the impact of environmental pollutants on lung diseases has also been a concern. Further research into whether environmental pollution is the main cause of lung diseases caused by glucometabolic reprogramming will help expand drug use. In addition, the dynamic nature of glucose metabolism in lung disease is a challenge for targeted drug therapy. In conclusion, reprogramming of glucose metabolism may be a key target for the prevention and treatment of lung disease, but further mechanistic studies are needed to investigate its specific principles.

## Glossary

ARDS: acute respiratory distress syndrome
ATP: adenosine triphosphate
AECs: alveolar epithelial cells
ATII: alveolar type II cells
AMs: alveolar macrophages
BAL: bronchoalveolar lavage
BLM: bleomycin
COPD: chronic obstructive pulmonary disease
CAP: community-acquired pneumonia
CAFs: cancer-associated fibroblasts
CAPM: COVID-19-associated pulmonary mucormycosis
CS COVID-19: cigarette smoke coronavirus disease 2019
DAD: diffuse alveolar damage
EMT: epithelial-mesenchymal transition
GLUTs: glucose transporters
GLUT1: glucose transporter 1
GDF15: growth differentiation factor 15
HHIP: hedgehog interacting protein
HIF-1: hypoxia-Inducible Factor 1 Complex
HIF-1α: hypoxia-inducible factor-1α
HRE: hypoxia response element
HK2: hexokinase 2
ICU: intensive care unit
JAK3: janus kinase
TR-AMs: tissue-resident alveolar macrophages
TREM-1: triggering receptor expressed on myeloid cells-1
TGF-β1: transforming growth factor-β1
TCA: LDH tricarboxylic acid lactate-by-lactate dehydrogenase
LPS: lipopolysaccharide
LUAD: lung adenocarcinoma
LUSC: lung squamous cell carcinoma
LIRI: lung ischemia-reperfusion injury
mTOR: mammalian target of rapamycin
MCT: monocarboxylate transporter
NSCLC: non-small cell lung cancer
NADH: nicotinamide adenine dinucleotide
NLRP3: NOD-like receptor protein 3
OXPHOS: oxidative phosphorylation
PDK1: pyruvate dehydrogenase kinase isozyme 1
PF: pulmonary fibrosis
PH: pulmonary hypertension
PPAR-γ: peroxisome proliferator-activated receptor γ
PFKFB3: 6-Phosphofructo-2-kinase/fructose-2, 6-bisphosphatase 3
PM2.5: PASMCs particulate matter pulmonary arterial smooth muscle cells
PKM2: pyruvate kinase M2
PIKK: phosphatidylinositol-3 kinase-related kinase
PTB: pulmonary tuberculosis
SCFA: short-chain fatty acid
SIRT3: sirtuin 3
SASPs: senescence-associated secretory phenotypes
USP54: ubiquitin-specific protease 54
